# Hypertensive disorders of pregnancy in Hunan Province, China, 2012–2022

**DOI:** 10.3389/fmed.2024.1415696

**Published:** 2024-12-20

**Authors:** Xu Zhou, Yinglan Wu, Xiaoying Chen, Yurong Jiang

**Affiliations:** Hunan Provincial Maternal and Child Health Care Hospital, Changsha, Hunan Province, China

**Keywords:** hypertensive disorders of pregnancy, adverse pregnancy outcomes, prevalence, risk factors, China

## Abstract

**Objective:**

To explore the relationship between hypertensive disorders of pregnancy (HDP) and adverse pregnancy outcomes and explore the risk factors for HDP.

**Methods:**

Data were obtained from the Maternal Near-Miss Surveillance System in Hunan Province, China, 2012–2022. Chi-square trend tests (*χ^2^*_trend_) were used to determine trends in prevalence by year. Unadjusted odds ratios (uORs) were used to examine the association between HDP and adverse pregnancy outcomes. Multivariate logistic regression analysis (method: Forward, Wald, *α* = 0.05) and adjusted odds ratios (aORs) were used to identify risk factors for HDP.

**Results:**

Our study included 780,359 pregnant women, and 38,397 women with HDP were identified, with a prevalence of 4.92% (95% CI 4.87–4.97). The prevalence of preeclampsia-eclampsia, gestational hypertension, chronic hypertension, and chronic hypertension with superimposed preeclampsia was 2.28% (95% CI 2.25–2.31), 2.04% (95% CI 2.00–2.07), 0.43% (95% CI 0.41–0.44), and 0.18% (95% CI 0.17–0.19), respectively. From 2012 to 2022, the prevalence of HDP increased from 3.11 to 7.39%, showing an upward trend (*χ*^2^_trend_ = 2220.88, *p* < 0.01). HDP was associated with the following adverse pregnancy outcomes: maternal deaths (uOR =4.05), maternal near-miss (uOR =6.37), preterm birth (uOR =2.51), stillbirth and neonatal death (uOR =1.45), low birthweight (uOR =4.37), abruptio placentae (uOR =4.45), uterine atony (uOR =1.49), retained placenta (uOR =1.54), puerperal infections (uOR =2.14), abdominal surgical site infections (uOR =2.50), urinary tract infections (uOR =1.60), upper respiratory tract infections (uOR =1.75), heart disease (uOR =2.76), embolism (uOR =2.66), liver disease (uOR =1.25), anemia (uOR =1.38), diabetes mellitus (uOR =2.35), renal disease (uOR =4.66), and pulmonary disease (uOR =4.70, *p* < 0.05). Results of multivariate logistic regression analysis showed risk factors for HDP: maternal age > 30 years (aOR > 1, *p* < 0.05), gravidity > = 4 (aOR =1.10, 95% CI 1.05–1.14), primipara (aOR > 1, *p* < 0.05), and previous cesarean sections (aOR =1.27, 95% CI 1.24–1.31).

**Conclusion:**

The prevalence of HDP was relatively high in Hunan Province. HDP was associated with many adverse pregnancy outcomes. Advanced maternal age, high gravidity, primipara, and previous cesarean section were risk factors for HDP.

## 1 Introduction

Hypertensive disorders of pregnancy (HDP) are a group of maternal disorders characterized by rising blood pressure during pregnancy, with systolic blood pressure > = 140 mm Hg and/or diastolic blood pressure > = 90 mm Hg (1). The global observed prevalence of HDP was 5.2–8.2% (2). The prevalence of HDP varies greatly by region. For example, the prevalence of HDP was 7.30% in China (a meta-analysis in 2021) (3), 7.44% in North China, 6.09% in Northeast China, 5.50% in Northwest China, 4.63% in East China, 3.20% in Southwest China, 2.59% in South China, and 1.23% in Central China (2011) (4). The prevalence of HDP was 10.6% in the United States (5), 6.73% in France (6), 8.5% in Denmark (7), 6.07% in Ethiopia (8), 8% in Sub-Saharan Africa (9).

HDP has many adverse outcomes for pregnant women and their fetuses. On the one hand, in the general population, hypertension is one of the leading causes of death due to heart disease and stroke (10). On the other hand, pregnant women are a special population, and if they suffer from HDP, it can be harmful not only to the pregnant woman herself but also to their fetuses. For example, Roberts et al. reported that women with HDP were five times more likely to have perinatal death compared to women without HDP (11); Say et al. reported that HDP was one of the leading causes of maternal and fetal deaths (12–14) and that it occurred mostly in developing countries (8). Garovic et al. reported that HDP was associated with adverse fetal outcomes, such as preterm delivery, small for gestational age, low birth weight, etc., (15). In addition, HDP may cause long-term adverse pregnancy outcomes. For example, it is well-accepted that the frequency of hypertension is significantly higher after HDP, and that hypertension develops faster than in pregnant women with normal blood pressure; The earlier onset of cardio-metabolic risk factors and cardiovascular disease events, as well as higher rates of accumulated chronic conditions and multi-morbidity, support the thesis of accelerated aging among women with a history of HDP (15); Boucheron et al. found that sustained HDP exposure was an additional risk factor for chronic hypertension (6); Mito et al. found that HDP was a strong risk factor for the development of hypertension only 5 years after delivery (16); Egawa et al. found HDP was associated with cardiovascular disease in middle- and older-aged Japanese women (17); Goldstein et al. reported that HDP was associated with future heart failure risk, including peripartum cardiomyopathy, pregnancy-associated heart failure with preserved ejection fraction, and new-onset heart failure later in life (18); Kanata et al. reported that HDP could have a significant clinical impacts not only on the mother’s but also on the offspring’s health (19–21).

Due to the high prevalence and adverse outcomes of HDP, research on HDP is very significant and deserves more attention. There have been some studies on HDP, such as epidemiology, prevention, diagnosis, and management (2, 3, 22–26). However, some research could be added to this field. First, to the best of our knowledge, there are fewer studies on HDP in Hunan Province, China. Hunan Province is located in south-central China and covers a population of about 65 million. Compared with eastern China, Hunan Province is relatively underdeveloped (27). As mentioned above, most HDP-related maternal and fetal deaths occurred in developing regions. Second, although there have been some studies on the prevalence of HDP in China, to the best of our knowledge, there are few studies on the relationship between HDP and adverse pregnancy outcomes and risk factors for HDP. More studies need to be included in China.

Therefore, in this study, we aim to describe the epidemiology of HDP, explore the relationship between HDP and adverse pregnancy outcomes, and explore the risk factors for HDP using long-term, large-area, and large-sample surveillance data from Hunan Province, China, 2012–2022. Our research will contribute to this field.

## 2 Methods

### 2.1 Data sources

This study used data from the Maternal Near-Miss Surveillance System in Hunan Province, China, 2012–2022, run by the Hunan Provincial Health Commission, and involves 18 representative registered hospitals in Hunan Province. These 18 hospitals are well-distributed throughout the province and are well-represented. In each of the 18 hospitals sampled, data were collected for all pregnant or post-partum women admitted to obstetrics departments. Surveillance data of all pregnant and post-partum women, including HDP, socio-demographic characteristics, obstetric history, and adverse pregnancy outcomes, were collected using an especially designed data collection form. Detailed information about the data collection process has been reported elsewhere (28). This study’s information collection methods and indicator definitions were consistent with the WHO standards (29, 30). In this study, the adverse pregnancy outcomes included maternal deaths, maternal near-miss, preterm birth, stillbirth and neonatal death, low birth weight, hemorrhage disorder, infections, and other common diseases (such as heart disease, embolism, liver disease, anaemia, diabetes mellitus, renal disease, and pulmonary disease).

### 2.2 Definition and classification of HDP

The definition and diagnosis of HDP complied with the 2021 International Society for the Study of Hypertension in Pregnancy classification, diagnosis, and management recommendations for international practice (1). According to previous studies, HDP is usually classified into four categories: (1) preeclampsia-eclampsia, (2) chronic hypertension, (3) chronic hypertension with superimposed preeclampsia, and (4) gestational hypertension (3, 31).

### 2.3 Ethics approval and consent to participate

The Hunan Provincial Health Commission routinely collected surveillance data, and the government has developed the “Maternal Near Miss Surveillance Working Manual” to collect those data. Therefore, there is no additional written informed consent. The Medical Ethics Committee of Hunan Provincial Maternal and Child Health Care Hospital approved the study (NO: 2024-S034). It is a retrospective study of medical records; all data were fully anonymized before we accessed them. Moreover, we de-identified the patient records before analysis. We confirmed that all operations were following relevant guidelines and regulations.

### 2.4 Data quality control

The Hunan Provincial Health Commission formulated the “Maternal Near Miss Surveillance Working Manual” for surveillance. Data were collected and reported by experienced and trained doctors and nurses. To ensure data consistency and accuracy, all collectors must be trained and qualified before starting work. The Hunan Provincial Health Commission asks the technical guidance departments to conduct comprehensive quality control yearly to reduce surveillance data integrity and information error rates.

### 2.5 Statistical analysis

We calculated the prevalence of HDP and 95% confidence intervals (CI) by the log-binomial method (32). Chi-square trend tests (*χ^2^*_trend_) were used to determine trends in prevalence by year. Unadjusted odds ratios (uORs) were used to examine the association between HDP and adverse pregnancy outcomes and demographic characteristics. Multivariate logistic regression analysis (method: Forward, Wald, *α* = 0.05) and adjusted odds ratios (aORs) were used to identify risk factors for HDP. We used the presence or absence of HDP as the dependent variable, and the demographic characteristics with significant uOR were entered as independent variables in multivariate logistic regression analysis.

Statistical analyses were performed using SPSS 18.0 (IBM Corp., NY, USA). Figures were drawn using GraphPad Prism 9.5 (GraphPad Software, MA, USA).

## 3 Results

### 3.1 Prevalence of HDP

Our study included 780,359 pregnant women, and 38,397 women with HDP were identified, with a prevalence of 4.92% (95% CI 4.87–4.97). Preeclampsia-eclampsia, gestational hypertension, chronic hypertension, and chronic hypertension with superimposed preeclampsia accounted for 46.31% (17,782 cases), 41.38% (15,889 cases), 8.70% (3,340 cases), and 3.61% (1,386 cases) of all HDP, respectively, and the prevalence was 2.28% (95% CI 2.25–2.31), 2.04% (95% CI 2.00–2.07), 0.43% (95% CI 0.41–0.44), and 0.18% (95% CI 0.17–0.19), respectively.

**Figure 1:**
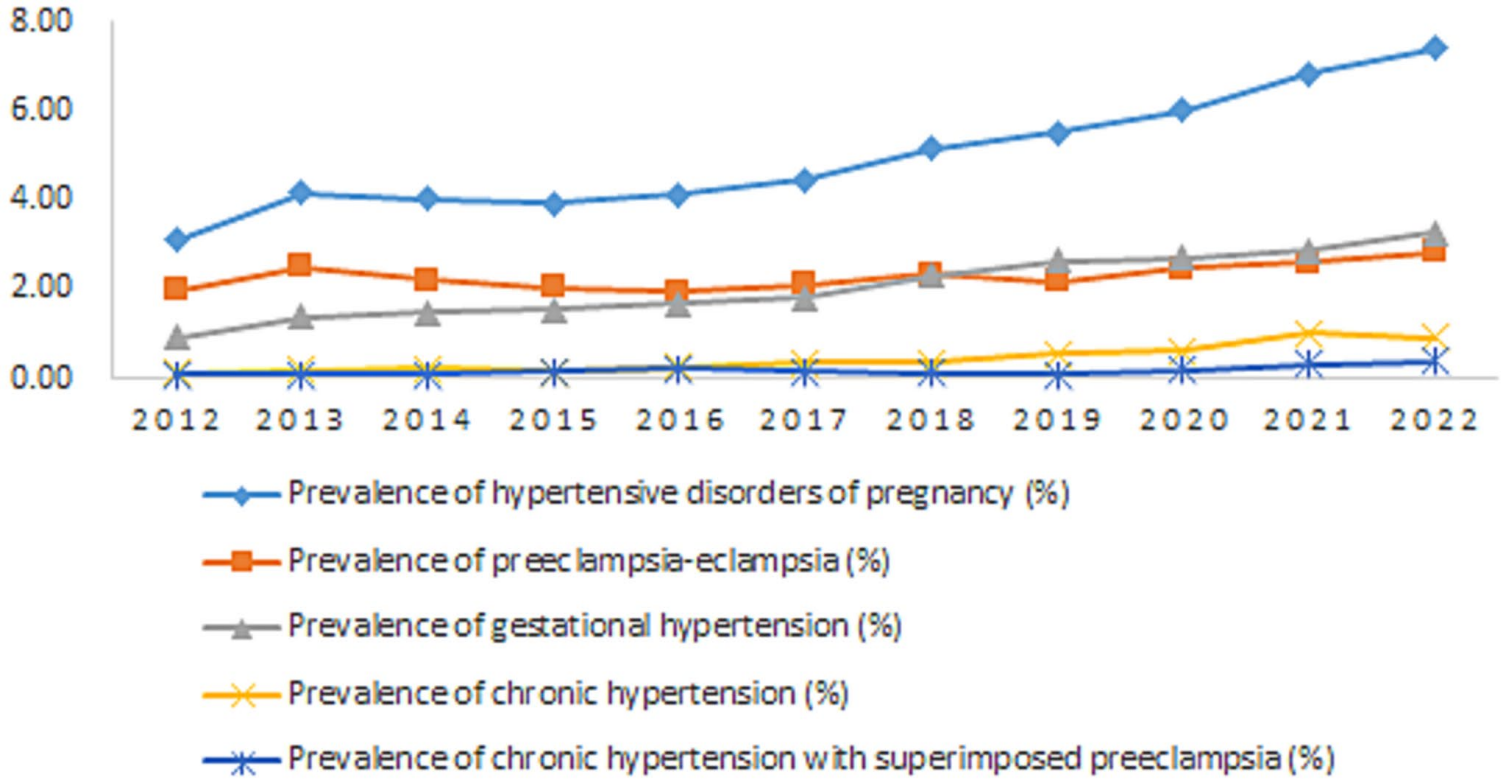
Prevalence of hypertensive disorders of pregnancy in Hunan Province, China, 2012–2022.

From 2012 to 2022, the prevalence of HDP increased from 3.11 to 7.39%, showing an upward trend (*χ*^2^_trend_ = 2220.88, *p* < 0.01); the prevalence of preeclampsia-eclampsia (*χ*^2^_trend_ = 97.37, *p* < 0.01), gestational hypertension (*χ*^2^_trend_ = 1738.97, *p* < 0.01), chronic hypertension (*χ*^2^_trend_ = 1184.67, *p* < 0.01), and chronic hypertension with superimposed preeclampsia (*χ*^2^_trend_ = 192.45, *p* < 0.01) also showed upward trends (Table 1 and Figure 1).

**Table 1:** Prevalence of hypertensive disorders of pregnancy in Hunan Province, China, 2012–2022.

Year	Pregnant women (*n*)	Hypertensive disorders of pregnancy (*n*)	Prevalence (%, 95 CI)	Subtypes							
				Preeclampsia-eclampsia (*n*)	Prevalence (%, 95 CI)	Gestational hypertension (*n*)	Prevalence (%, 95 CI)	Chronic hypertension (*n*)	Prevalence (%, 95 CI)	Chronic hypertension with superimposed preeclampsia (*n*)	Prevalence (%, 95 CI)
2012	62,608	1944	3.11 (2.97–3.24)	1,229	1.96 (1.85–2.07)	574	0.92 (0.84–0.99)	81	0.13 (0.10–0.16)	60	0.10 (0.07–0.12)
2013	67,886	2,825	4.16 (4.01–4.31)	1,691	2.49 (2.37–2.61)	933	1.37 (1.29–1.46)	137	0.20 (0.17–0.24)	64	0.09 (0.07–0.12)
2014	75,817	3,053	4.03 (3.88–4.17)	1,674	2.21 (2.10–2.31)	1,131	1.49 (1.40–1.58)	165	0.22 (0.18–0.25)	83	0.11 (0.09–0.13)
2015	79,513	3,127	3.93 (3.79–4.07)	1,629	2.05 (1.95–2.15)	1,223	1.54 (1.45–1.62)	142	0.18 (0.15–0.21)	133	0.17 (0.14–0.20)
2016	71,310	2,950	4.14 (3.99–4.29)	1,387	1.95 (1.84–2.05)	1,202	1.69 (1.59–1.78)	197	0.28 (0.24–0.31)	164	0.23 (0.19–0.27)
2017	78,919	3,518	4.46 (4.31–4.61)	1,673	2.12 (2.02–2.22)	1,437	1.82 (1.73–1.92)	287	0.36 (0.32–0.41)	121	0.15 (0.13–0.18)
2018	76,647	3,943	5.14 (4.98–5.30)	1804	2.35 (2.25–2.46)	1763	2.30 (2.19–2.41)	283	0.37 (0.33–0.41)	93	0.12 (0.10–0.15)
2019	75,033	4,127	5.50 (5.33–5.67)	1,636	2.18 (2.07–2.29)	1984	2.64 (2.53–2.76)	427	0.57 (0.52–0.62)	80	0.11 (0.08–0.13)
2020	68,756	4,120	5.99 (5.81–6.18)	1,695	2.47 (2.35–2.58)	1860	2.71 (2.58–2.83)	427	0.62 (0.56–0.68)	138	0.20 (0.17–0.23)
2021	64,101	4,371	6.82 (6.62–7.02)	1,678	2.62 (2.49–2.74)	1839	2.87 (2.74–3.00)	647	1.01 (0.93–1.09)	207	0.32 (0.28–0.37)
2022	59,769	4,419	7.39 (7.18–7.61)	1,686	2.82 (2.69–2.96)	1943	3.25 (3.11–3.40)	547	0.92 (0.84–0.99)	243	0.41 (0.36–0.46)
Total	780,359	38,397	4.92 (4.87–4.97)	17,782	2.28 (2.25–2.31)	15,889	2.04 (2.00–2.07)	3,340	0.43 (0.41–0.44)	1,386	0.18 (0.17–0.19)

CI, confidence intervals.

### 3.2 Relationship between HDP and adverse pregnancy outcomes

**Figure 2:**
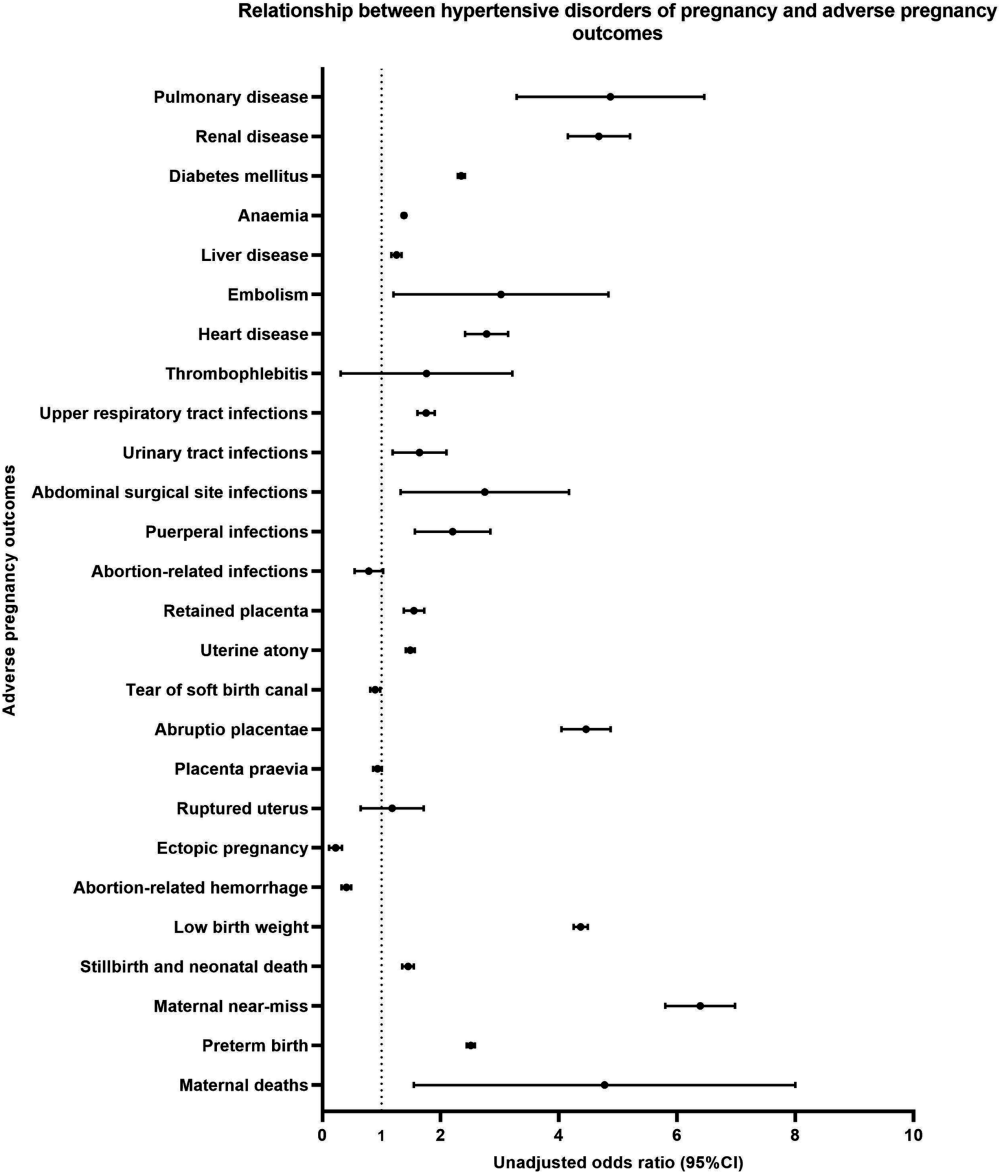
Relationship between hypertensive disorders of pregnancy and adverse pregnancy outcomes.

HDP was associated with the following adverse pregnancy outcomes: maternal deaths (uOR =4.05), maternal near-miss (uOR =6.37), preterm birth (uOR =2.51), stillbirth and neonatal death (uOR =1.45), low birthweight (uOR =4.37), abruptio placentae (uOR =4.45), uterine atony (uOR =1.49), retained placenta (uOR =1.54), puerperal infections (uOR =2.14), abdominal surgical site infections (uOR =2.50), urinary tract infections (uOR =1.60), upper respiratory tract infections (uOR =1.75), heart disease (uOR =2.76), embolism (uOR =2.66), liver disease (uOR =1.25), anemia (uOR =1.38), diabetes mellitus (uOR =2.35), renal disease (uOR =4.66), and pulmonary disease (uOR =4.70, *p* < 0.05) (Table 2 and Figure 2).

**Table 2:** Relationship between hypertensive disorders of pregnancy and adverse pregnancy outcomes.

Adverse pregnancy outcomes	Proportion in HDP	Proportion in no-HDP	uOR (95% CI)
Maternal deaths	0.02% (9 / 38,397)	0.01% (43 / 741,962)	4.05 (1.97–8.30)
Maternal near-miss	1.57% (604 / 38,397)	0.25% (1857 / 741,962)	6.37 (5.81–6.99)
Preterm birth	19.53% (7,498 / 38,397)	8.82% (65,433 / 741,962)	2.51 (2.44–2.58)
Stillbirth and neonatal death	2.38% (913 / 38,397)	1.66% (12,293 / 741,962)	1.45 (1.35–1.55)
Low birth weight	18.74% (7,194 / 38,397)	5.01% (37,183 / 741,962)	4.37 (4.25–4.49)
Hemorrhage disorder			
Abortion-related hemorrhage	0.25% (96 / 38,397)	0.62% (4,604 / 741,962)	0.40 (0.33–0.49)
Ectopic pregnancy	0.04% (15 / 38,397)	0.19% (1,411 / 741,962)	0.21 (0.12–0.34)
Ruptured uterus	0.05% (19 / 38,397)	0.04% (333 / 741,962)	1.10 (0.69–1.75)
Placenta praevia	1.58% (608 / 38,397)	1.70% (12,579 / 741,962)	0.93 (0.86–1.01)
Abruptio placentae	1.42% (545 / 38,397)	0.32% (2,395 / 741,962)	4.45 (4.05–4.88)
Tear of soft birth canal	1.18% (454 / 38,397)	1.32% (9,809 / 741,962)	0.89 (0.81–0.98)
Uterine atony	4.45% (1707 / 38,397)	3.03% (22,518 / 741,962)	1.49 (1.41–1.56)
Retained placenta	0.83% (317 / 38,397)	0.54% (3,979 / 741,962)	1.54 (1.38–1.73)
Infections			
Abortion-related infections	0.11% (42 / 38,397)	0.14% (1,066 / 741,962)	0.76 (0.56–1.04)
Puerperal infections	0.13% (50 / 38,397)	0.06% (452 / 741,962)	2.14 (1.60–2.87)
Abdominal surgical site infections	0.04% (15 / 38,397)	0.02% (116 / 741,962)	2.50 (1.46–4.28)
Urinary tract infections	0.14% (53 / 38,397)	0.09% (640 / 741,962)	1.60 (1.21–2.12)
Upper respiratory tract infections	1.65% (632 / 38,397)	0.95% (7,027 / 741,962)	1.75 (1.61–1.90)
Thrombophlebitis	0.01% (5 / 38,397)	0.01% (71 / 741,962)	1.36 (0.55–3.37)
Other common diseases			
Heart disease	0.65% (250 / 38,397)	0.24% (1757 / 741,962)	2.76 (2.42–3.15)
Embolism	0.03% (11 / 38,397)	0.01% (80 / 741,962)	2.66 (1.41–4.99)
Liver disease	2.55% (980 / 38,397)	2.05% (15,206 / 741,962)	1.25 (1.17–1.34)
Anaemia	30.78% (11,819 / 38,397)	24.35% (180,662 / 741,962)	1.38 (1.35–1.41)
Diabetes mellitus	20.33% (7,806 / 38,397)	9.81% (72,784 / 741,962)	2.35 (2.29–2.41)
Renal disease	0.98% (378 / 38,397)	0.21% (1,581 / 741,962)	4.66 (4.16–5.21)
Pulmonary disease	0.11% (44 / 38,397)	0.02% (181 / 741,962)	4.70 (3.38–6.54)

HDP, hypertensive disorders of pregnancy; uOR, unadjusted odds ratio; CI, confidence intervals.

### 3.3 Epidemiology of and multivariate logistic regression analysis for risk factors for HDP

Table 3 shows the epidemiology of HDP. Univariate analysis shows that all variables in Table 3 are associated with HDP. Therefore, all variables in Table 3 were used as independent variables in the multivariate logistic regression analysis. As a result, all variables in Table 3 entered the regression model.

**Table 3:** Epidemiology of and multivariate logistic regression analysis for risk factors for hypertensive disorders of pregnancy.

Demographic characteristics	Pregnant women (*n*)	Hypertensive disorders of pregnancy (*n*)	Prevalence (%, 95 CI)	uOR (95%CI)	aOR (95% CI)
Maternal age (years old)					
25–29	313,178	12,152	3.88 (3.81–3.95)	Reference	Reference
<20	9,689	235	2.43 (2.12–2.74)	0.62 (0.54–0.70)	0.60 (0.53–0.69)
20–24	112,206	3,718	3.31 (3.21–3.42)	0.85 (0.82–0.88)	0.84 (0.81–0.87)
30–34	234,862	12,737	5.42 (5.33–5.52)	1.42 (1.38–1.46)	1.54 (1.50–1.58)
> = 35	110,424	9,555	8.65 (8.48–8.83)	2.35 (2.28–2.41)	2.77 (2.69–2.86)
Gravidity					
1	269,127	13,524	5.03 (4.94–5.11)	Reference	Reference
2	228,821	9,887	4.32 (4.24–4.41)	0.85 (0.83–0.88)	0.95 (0.92–0.98)
3	144,195	6,928	4.80 (4.69–4.92)	0.95 (0.93–0.98)	1.01 (0.97–1.05)
> = 4	138,216	8,058	5.83 (5.70–5.96)	1.17 (1.14–1.20)	1.10 (1.05–1.14)
Parity (Not including this time)					
0 (Primipara)	400,250	20,982	5.24 (5.17–5.31)	Reference	Reference
1	328,532	14,730	4.48 (4.41–4.56)	0.85 (0.83–0.87)	0.56 (0.54–0.58)
> = 2	51,577	2,685	5.21 (5.01–5.40)	0.99 (0.95–1.03)	0.59 (0.56–0.62)
Prenatal examination (times)					
8–10	401,909	22,439	5.58 (5.51–5.66)	Reference	Reference
<5	103,946	3,510	3.38 (3.27–3.49)	0.59 (0.57–0.61)	0.67 (0.65–0.70)
5–7	222,708	9,681	4.35 (4.26–4.43)	0.77 (0.75–0.79)	0.86 (0.84–0.88)
> = 11	51,796	2,767	5.34 (5.14–5.54)	0.95 (0.92–0.99)	0.98 (0.94–1.02)
Previous cesarean section					
No	634,941	30,283	4.77 (4.72–4.82)	Reference	Reference
Yes	145,418	8,114	5.58 (5.46–5.70)	1.18 (1.15–1.21)	1.27 (1.24–1.31)

uOR, unadjusted odds ratio; CI, confidence intervals; aOR, adjusted odds ratio.

Compared to maternal age 25–29 years, HDP were less common in <20 years (aOR =0.60, 95% CI 0.53–0.69) or 20–24 years (aOR =0.84, 95% CI 0.81–0.87) and more common in 30–34 years (aOR =1.54, 95% CI 1.50–1.58) or > =35 years (aOR =2.77, 95% CI 2.69–2.86). Compared to the gravidity = 1, HDP were less common in gravidity = 2 (aOR =0.95, 95% CI 0.92–0.98) and more common in gravidity > = 4 (aOR =1.10, 95% CI 1.05–1.14). Compared to parity = 0, HDP were less common in parity = 1 (aOR =0.56, 95% CI 0.54–0.58) or > =2 (aOR =0.59, 95% CI 0.56–0.62). Compared to prenatal examination = 8–10 times, HDP were less common in prenatal examination <5 times (aOR =0.67, 95% CI 0.65–0.70) or 5–7 times (aOR =0.86, 95%CI 0.84–0.88). HDP was more common in previous cesarean sections than vaginal delivery (aOR =1.27, 95% CI 1.24–1.31).

**Figure 3:**
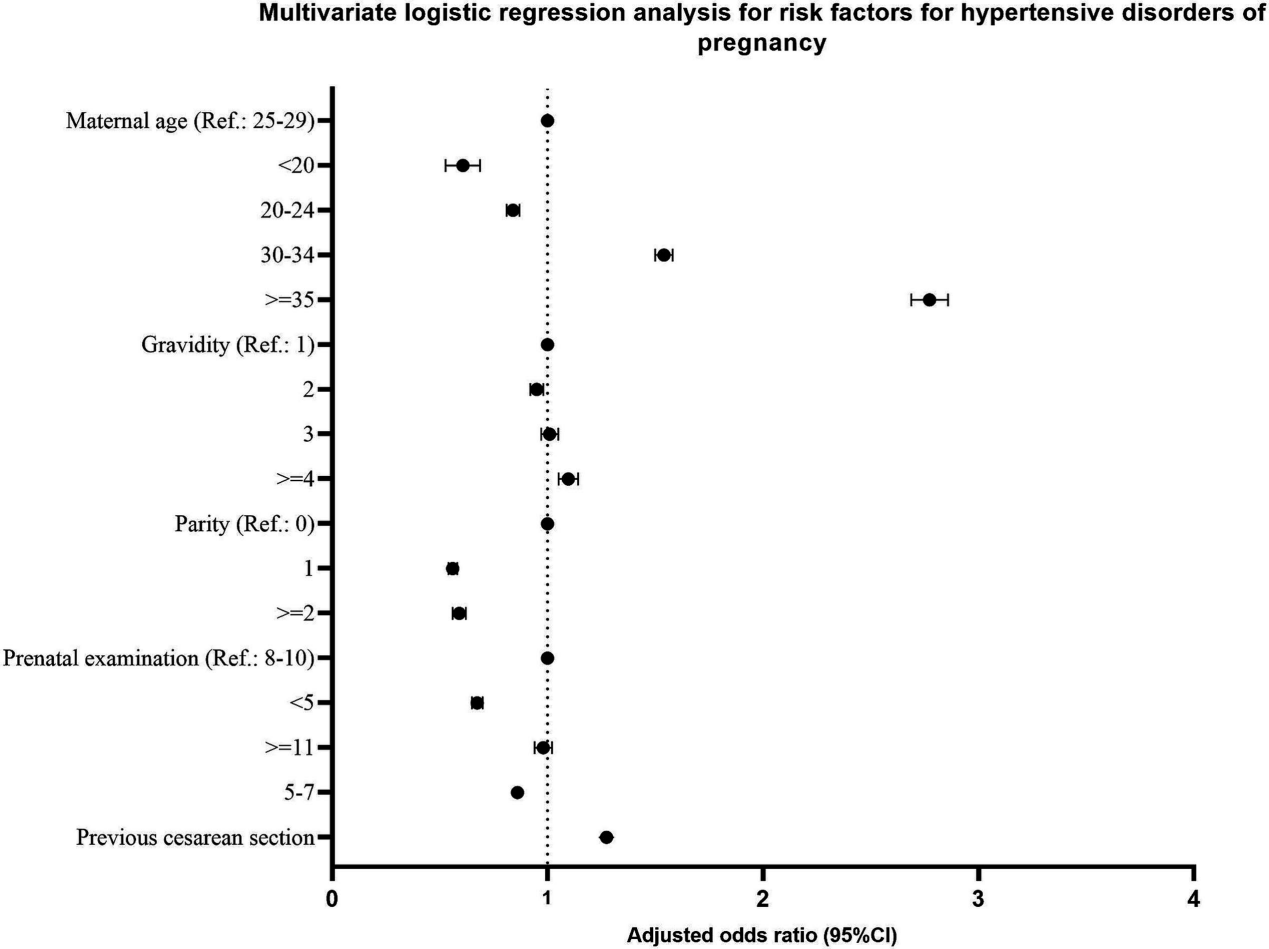
Multivariate logistic regression analysis for risk factors for hypertensive disorders of pregnancy.

There were significant differences in the results of univariate analysis and multivariate logistic regression analysis for some factors. For example, gravidity = 3 was a protective factor for HDP in the univariate analysis (uOR =0.95, 95% CI 0.93–0.98), while not in the multivariate logistic regression analysis (aOR =1.01, 95% CI 0.97–1.05). Parity > = 2 was a protective factor for HDP in the multivariate logistic regression analysis (aOR =0.59, 95% CI 0.56–0.62), while not in the univariate analysis (uOR =0.99, 95% CI 0.95–1.03). Prenatal examination > = 11 times was a protective factor for HDP in the univariate analysis (uOR =0.95, 95%CI 0.92–0.99), while not in the multivariate logistic regression analysis (aOR =0.98, 95% CI 0.94–1.02) (Table 3 and Figure 3).

## 4 Discussion

Overall, we described the epidemiology of HDP, explored the relationship between HDP and adverse pregnancy outcomes, and explored risk factors for HDP. Our study is the most recent systematic study of the relationship between adverse pregnancy outcomes, risk factors, and HDP in China. Our research makes some original contributions to the field. There are several relevant findings in this study.

First, the prevalence of HDP (average: 4.92%; 2022: 7.39%) in this study was relatively high, which led to a considerable number of pregnant women being affected by HDP. The global prevalence of HDP was 5.2–8.2%, and the average prevalence in China was 7.30% (2, 3), which is consistent with the 2022 prevalence in this study. However, as mentioned in the Introduction, there are significant differences in the prevalence of HDP in different countries or regions. For example, Ye et al. reported that the prevalence of HDP was 7.44% in North China, 6.09% in Northeast China, 5.50% in Northwest China, 4.63% in East China, 3.20% in Southwest China, 2.59% in South China, and 1.23% in Central China (2011) (4). Hunan Province is located in south-central China, and the prevalence of HDP in this study was significantly higher than in Ye’s study. In addition, the prevalence of HDP in this study showed a significant upward trend from 3.11% in 2012 to 7.39% in 2022. It is inconsistent with the decreasing trend in most countries and regions (33). The above findings may be associated with several factors. First, differences in the prevalence of HDP in different countries may be primarily related to variants in race, economics, and medical conditions. For example, Ward et al. found genetic variants related to HDP in different races (34, 35). Due to limitations in economic and medical conditions, some less developed countries have relatively low HDP diagnosis rates (36–38), and fewer studies have been conducted, or only in a few hospitals, in a small area, and in a long time ago, which may be unrepresentative and lead to biased results (4, 39–42). Second, the differences in the prevalence of HDP in different regions of China may be primarily related to variants in lifestyle, economic, and medical conditions. For example, Lu et al. found regional disparities in prenatal care and socio-economic in China (43, 44), which may affect the diagnosis and treatment of HDP. Unhealthy urban lifestyles (e.g., alcohol drinking and late-night eating) exist in Hunan, which may increase the risk of HDP (45). Third, the upward trend in the prevalence of HDP may indicate an increase in some risk factors and may also be partly related to higher diagnosis rates due to improved medical conditions. For example, Umesawa et al. reported that the risk factors for HDP included body mass index, anemia, smoking, alcohol intake, education, maternal age, primipara, previous experience of pregnant complications, gestational diabetes mellitus, preexisting disease (such as diabetes mellitus), urinary tract infection, family history, and genetic variants (2). In recent years, the prevalence of many risk factors has increased, such as overweight and obesity (46), advanced maternal age (47, 48), and diabetes mellitus (49, 50). Especially after the implementation of the “two-child policy” in 2015, many risk factors have increased significantly (51, 52). It may be the main reason for the upward trend of HDP prevalence in this study. In addition, as mentioned above, with the development of medical conditions (27), the diagnosis rate of HDP is gradually increasing, which may also be one of the reasons for the upward trend of HDP prevalence.

In this study, we also reported the prevalence of preeclampsia-eclampsia (2.28%), gestational hypertension (2.04%), chronic hypertension (0.43%), and chronic hypertension with superimposed preeclampsia (0.18%). Li et al. reported that the prevalence of preeclampsia-eclampsia, gestational hypertension, chronic hypertension, and chronic hypertension with superimposed preeclampsia were 4.50, 3.30, 0.60, and 0.60%, respectively (China) (3). Umesawa et al. reported the prevalence of gestational hypertension was 1.8–4.4% (global) (2). The American College of Obstetricians and Gynecologists reported that chronic hypertension is present in 0.9–1.5% of pregnant women (53), and preeclampsia complicates 2–8% of pregnancies globally (54). In this study, the prevalence of chronic hypertension and chronic hypertension with superimposed preeclampsia was relatively low. It may be partly associated with missed or misdiagnosed cases, and this phenomenon is more common in areas with poor medical conditions. To the best of our knowledge, some previous studies of HDP in China did not provide a detailed classification of subtypes, and some did not use internationally recognized classification methods. In this study, the internationally recognized classification of HDP was used for the first time in Hunan Province, allowing for a comparison of the prevalence in different regions.

Second, we found that HDP was associated with many adverse pregnancy outcomes. Previous studies have shown that HDP was associated with many adverse pregnancy outcomes, such as maternal deaths (12, 13), maternal near-miss (55), low birthweight and preterm birth (56), stillbirth, neonatal death (57, 58), abruptio placentae (59), retained placenta (60), puerperal infections (61), urinary tract infections (62), liver disease (63), and pulmonary disease (64). However, some of the adverse pregnancy outcomes in this study had been rarely addressed in previous studies, such as uterine atony, abdominal surgical site infections, upper respiratory tract infections, embolism, anemia, and connective tissue disease. In addition, this study provided the prevalence and risk values (ORs) of HDP in different populations. It may be helpful in clinical counseling. This is an observational study, and we cannot determine whether there is a causal relationship between HDP and adverse pregnancy outcomes. HDP may contribute to some adverse pregnancy outcomes, while some adverse pregnancy outcomes may be responsible for HDP. In-depth studies are needed to explore the mechanisms. This study is helpful for future research.

Third, we identified several risk factors for HDP, including advanced maternal age, high gravidity, primipara, and previous cesarean section, and low-frequency prenatal examination was the only protective factor for HDP. Some risk factors have been widely accepted, such as advanced maternal age, primipara, and cesarean section (2, 65). However, to the best of our knowledge, previous studies rarely addressed some factors, such as high gravidity and low-frequency prenatal examination. We infer that the high gravidity may be associated with some pregnancy complications, such as recurrent miscarriages (66), which may be associated with HDP (67, 68). The low-frequency prenatal examination may be mainly associated with poor economic and medical conditions and low education, which may cause a relatively low diagnosis rate of HDP. It has been discussed above. Similar to the relationship between HDP and adverse pregnancy outcomes mentioned above, the relationship between HDP and the above factors could not be determined to be causal.

Overall, the prevalence of HDP was relatively high in Hunan Province, and we have identified several risk factors for HDP. It may contribute to public health interventions or prenatal care strategies. For example, to avoid HDP, we advise that women try to avoid getting pregnant at an advanced age. The government can implement public health programs for early diagnosis and free treatment of HDP among pregnant women with high gravidity or previous cesarean section, or primiparous women, to reduce the patients’ burden.

This study could have been improved. First, due to data limitations, some factors, such as body mass index, lifestyle, economic conditions, education, family history, and race, were not included. Second, this was an observational study, and we could not determine whether there was a causal association between HDP and adverse pregnancy outcomes and demographic characteristics. Third, the prevalence of HDP may be slightly underestimated. This study is helpful for future research.

## 5 Conclusion

The prevalence of HDP was relatively high in Hunan Province. HDP was associated with many adverse pregnancy outcomes. Advanced maternal age, high gravidity, primipara, and previous cesarean section were risk factors for HDP.

## Data Availability

The original contributions presented in the study are included in the article/supplementary material, further inquiries can be directed to the corresponding authors.
